# Criminal Prohibition of Wrongful Re‑identification: Legal Solution or Minefield for Big Data?

**DOI:** 10.1007/s11673-017-9806-9

**Published:** 2017-09-14

**Authors:** Mark Phillips, Edward S. Dove, Bartha M. Knoppers

**Affiliations:** 1 Centre of Genomics and Policy, Faculty of Medicine, McGill University, 740, Dr. Penfield Avenue, Room 5209, Montreal, Quebec H3A 0G1 Canada; 2 J. Kenyon Mason Institute for Medicine, Life Sciences and the Law, School of Law, University of Edinburgh, Old College, South Bridge, Edinburgh, EH8 9YL UK; 3 Centre of Genomics and Policy, Faculty of Medicine, McGill University, 740, Dr. Penfield Avenue, Room 5214, Montreal, Quebec H3A 0G1 Canada

**Keywords:** Anonymization, Re-identification, Data protection, Criminal law, Big data, Medicine

## Abstract

The collapse of confidence in anonymization (sometimes also known as de-identification) as a robust approach for preserving the privacy of personal data has incited an outpouring of new approaches that aim to fill the resulting trifecta of technical, organizational, and regulatory privacy gaps left in its wake. In the latter category, and in large part due to the growth of Big Data–driven biomedical research, falls a growing chorus of calls for criminal and penal offences to sanction wrongful re-identification of “anonymized” data. This chorus cuts across the fault lines of polarized privacy law scholarship that at times seems to advocate privacy protection at the expense of Big Data research or vice versa. Focusing on Big Data in the context of biomedicine, this article surveys the approaches that criminal or penal law might take toward wrongful re-identification of health data. It contextualizes the strategies within their respective legal regimes as well as in relation to emerging privacy debates focusing on personal data use and data linkage and assesses the relative merit of criminalization. We conclude that this approach suffers from several flaws and that alternative social and legal strategies to deter wrongful re-identification may be preferable.

## Introduction

A new form of data protection prohibition is arriving. Since 2010, a range of scholars and lawmakers, particularly in the biomedical context, have gravitated toward calling for criminal penalties for wrongful re-identification of anonymized data.

This trend has three striking features. First, it has emerged rapidly and simultaneously across numerous jurisdictions around the globe, primarily in response to damaging reports of new data breaches and of re-identification of data that had been presumed to have been adequately anonymized. Second, in contrast with other recent data protection legislative trends, such as the adoption of breach notification rules, re-identification criminalization appears to be a less-natural extension of fundamental data protection principles. Instead, it represents a new principle overlapping significantly with elements of the existing data protection framework. Third, existing scholarly and legislative treatment of the topic have been cursory, likely because it was catalyzed in response to individual crises or patterns thereof, rather than emanating from the theoretical or empirical evolution of data protection.

This article advances this discussion through a sustained, critical appraisal of criminal re-identification penalties. Because the trend is international, the analysis is comparative. A measure of specificity is inevitably lost as data protection regimes, although they share important common features, do vary significantly internationally, much like the broader legal frameworks within which they are inscribed. But the underlying issues in question go to the conceptual heart of data protection, especially the nature of the relationship between the technical and legal means of data protection—in particular how one might be substituted for the other—a topic recently developed in the *Breyer* case in Europe that found dynamic IP addresses may qualify as personal data (*Breyer v Bundesrepublik Deutschland* (C-582/14) [2016] ECJ).

The analysis follows in six parts. This article first relates the developments underpinning the wave of interest in criminal penalties for wrongful re-identification. It next identifies diverse proponents that these new penalties have attracted. Third, it examines explicit regulation of re-identification in general, abstracted from the criminal or other nature of the enforcement mechanism. Fourth, it analyses the scope or breadth of actors and activities to which the sanction can apply. Fifth, it discusses the role of the intensity of the criminal sanction in the data protection context in general and in the re-identification context in particular. Sixth, it analyses a recent legislative bill that would explicitly criminalize re-identification, which was introduced in Australia at the end of 2016 following a scandal involving the re-identification of clinical data published for research purposes. Finally, we conclude with our assessment of this criminalization trend, noting that at this time its drawbacks likely outweigh its putative benefits, and ultimately advocate alternative strategies such as community sanctions or punitive damages or fines calibrated for deterrence.

## I. Catalysts

Numerous voices are calling for the criminalization of wrongful re-identification to enable health benefits through Big Data analysis (e.g. National Data Guardian for Health and Care 2016; Pilgrim 2016; Gellman 2011; Nass, Levit, and Gostin 2009). The idea is to provide a convincing answer to the privacy concerns that might otherwise compromise medical breakthroughs enabled by analysing large or linked datasets of personal data in order to ensure that these benefits are neither passed up nor even delayed. Criminalization advocates have not built their case by providing examples of important, real-life medical advances that could only have been achieved through Big Data analysis nor have they described how any potential benefits will be distributed between different social groups and classes. Rather, they tend to refer to missed speculative breakthroughs and opportunities.

This “Big Data Benefits” argument is nonetheless backed by considerable moral force. Scholars who fall strongly on the side of privacy protection even appear prepared to carve out exceptions where health research is at stake, such as leading security expert Bruce Schneier (Schneier 2015). In the law and policy field, Big Data Benefits advocates have also recently set their sights on transforming this moral force into a legal one. The human right “to share in scientific advancement and its benefits,” as set forth in article 27 of the *Universal Declaration of Human Rights*, is being interpreted in the literature as facilitating researcher access to health-related personal data to further operationalize the open data and open science movements (Knoppers et al. 2014). Indeed, those deemed to unduly restrict access to data, particularly those generated through public funds, may be seen as “data hoarders” undermining the values of solidarity and altruism (Prainsack 2015).

Improving human health is as uncontroversial an objective as one is likely to encounter. In weighing benefits and risks when deciding how to go about doing so, a discussion that remains conspicuously unexplored, however, is the people to whom the benefits of any given research initiative flow in particular. Does investment in a given emerging healthcare technology or strategy (personalized medicine, for instance) increase or decrease existing disparities in healthcare quality or access? Does it increase or decrease the efficiency and proportion of benefits that flow to the public-at-large in relation to private sector actors such as insurers and drug companies?

But the primary countervailing considerations where Big Data medical science is concerned—and the ones this article discusses—are privacy and data protection,^1^ which have taken on a fundamental importance and are the object of increased anxiety. In terms of security, for example, “[i]n 2015, the US health sector was affected by some of the largest data breaches in its history,” in which over 100 million healthcare records were stolen (Australian Government Productivity Commission 2016, 223). The number of patients no longer willing to freely disclose their medical information to healthcare providers for fear of such breaches has rapidly increased (Black Book Market Research 2017). The perceived economic value of Big Data may now be in decline as a result of the economic consequences of these incidents. There is increased potential for Big Data to become a “toxic asset,” whose value is optimized by erasing it once the purpose for which it has been collected has been fulfilled (Schneier 2016), including when it remains stored yet largely unexploited (“dark data”).

In 2015 and 2016, hospitals were also targeted specifically by ransomware exploits that not only extracted large payments from the institutions by blackmail but that also caused incidental service outages (Hennigan and Bennett 2016). Ransomware has since been made illegal in California, where many of these incidents occurred (Fisher 2017). Though it is difficult to obtain accurate statistics—companies are loath to publicly discuss having been compromised—an IBM study found that 70 per cent of businesses infected by ransomware made payments as a means to restore their access to their own systems (IBM 2016).

Actors in the informal economy have no monopoly on privacy concerns. The broad scope of indiscriminate surveillance by much more powerful actors, governments and corporations, of course, was confirmed by Edward Snowden’s revelations in 2012. Risks including these have led to intensified concerns from patients and research participants about secondary sharing of their data (Robertson 2013).

Until recently, the tension between robust data protection and Big Data Benefits was nonetheless felt to be reconcilable without much compromise. Because the effectiveness of scientific and Big Data analysis do not generally depend on the individual identities of their subjects, by anonymizing the data—that is, irreversibly disassociating the subjects’ identities—the leading current of thought held that use and disclosure could be free of any significant risk of adverse privacy consequences.^2^ Even in the context of rich, multidimensional data, advocates could once be found favouring the idea that genomic data is anonymized when no direct identifiers are included, such as a person’s name or telephone number (Nietfeld 2007; Lowrance 2002, 34).

But practical anonymization has proven more elusive than hoped. Latanya Sweeney’s pioneering work demonstrating the ease of re-identifying “de-identified” research participants (Sweeney 2000), was successively built upon by an array of clever research, which was surveyed in the landmark legal study of the question by Paul Ohm (Ohm 2010). These revelations have mortally wounded the conception of anonymization as a robust privacy-preserving approach.

Genomic re-identification experts, in particular, have demonstrated a series of surprising results including the ability to use aggregated genetic variant data to determine whether or not a given research participant had the disease being studied (Homer et al. 2008), to link a person’s bioinformatic profile with their name in an online genealogy database (Gymrek et al. 2013), and to re-identify a research participant using only 25 of their genetic variants, selected at random (Cai et al. 2015).

In light of these recent discoveries, anonymization’s defenders have had to reassess their claims. A measure of debate—at times fierce—has persisted about the continued relevance of anonymization (El Emam and Arbuckle 2014; Sweeney 2015). This divergence of experts should itself serve as a caution against assertions of reliable forecasts of re-identification risk forecasts, apart from when using specific measurable techniques such as differential privacy, which adds noise to data until it is unidentifiable according to a certain threshold metric. Anonymization’s detractors have been more categorical in asserting that “the privacy risk of data that is protected by ad hoc de-identification is not just unknown, but unknowable” (Narayanan, Huey, and Felten 2015, 1) and that ultimately “[a]nonymity does not solve ethical problems relative to privacy in a big data age” (Barocas and Nissenbaum 2014, 51).

The inevitable technical uncertainty has been needlessly exacerbated by a proliferation of technical terms to describe data identifiability and the adoption of contradictory definitions by a variety of entities (Phillips and Knoppers 2016). Among the still-all-too-common consequences are, on the one hand, patently inadequate “anonymization,” such as merely removing names while leaving home addresses intact (McLean 2016), and, on the other, needless barriers to medical researchers’ access to data sets for analysis (Council of Canadian Academies 2015).

The discussion of the recent explosion of healthcare data breaches mentioned above needs to be qualified when we come to a discussion of the health research sector, however. To date, extremely few breaches by malicious actors have come to light (Laurie et al. 2014). Publicized re-identification attacks seem to be limited to those developed by security researchers seeking to identify weaknesses in anonymization techniques in order to strengthen data protection, rather than to exploiting the weaknesses to the detriment of research participants (Yakowitz 2015). Rogue actors in the informal economy, at least for the moment, prefer access to identified, raw data and to large clinical data sets. But incentives and targets can change quickly. For the moment the effort required to breach research data sets seems not to be worth the effort, but if the healthcare data repositories that are currently besieged substantially increase their security practices, research data sets may suddenly become the most compelling health data target.

## II. Proponents of Criminalizing Wrongful Re-identification

The weaknesses of traditional anonymization techniques have incited a flurry of activity seeking to craft alternative technical, organizational, and regulatory data protection solutions. This article focuses on one type of proposed solution, namely criminal sanctions against inappropriate re-identification.

As mentioned in the introduction, a striking feature of this trend is the variety of adherents it has attracted. Discussions about privacy and Big Data include both privacy protectors on one side and promoters of Big Data Benefits on the other. Yet, advocates of criminalizing re-identification transcend the two sides of this debate.

The earliest explicit published call for such penalties may be Ohm’s 2010 landmark review of anonymization, which made only a cursory call in response to the identified shortcomings for “additional safeguards and accountability mechanisms … [f]or example … new sanctions—possibly even criminal punishment—for those who reidentify” (Ohm 2010, 1770).

A more sustained proposal came the following year (Gellman 2011), when privacy consultant Robert Gellman drew on the earlier *Beyond the HIPAA Privacy Rule* report, which had advocated for “legal sanctions [generally] to prohibit unauthorised reidentification” (Nass et al. 2009, 281). That report in turn drew its inspiration (Nass et al. 2009) from a report commissioned by the U.K. Prime Minister on secondary use (Thomas and Walport 2008)*.*

Gellman’s article included model statutory provisions (Gellman 2011, 55–61) that would create a felony offence for wilful or attempted re-identification. His offence applies only to entities^3^ that enter into a data-access agreement in which they explicitly and irrevocably consent to accept the potential to be held liable for the offence. The provisions include other data protection safeguards, such as requiring the entity to “promptly report any breach of [such] a data agreement” (Gellman 2011, 58). Daniel Barth-Jones, an infectious disease epidemiologist, soon reiterated the aspiration to see criminal re-identification penalties passed in the United States (Barth-Jones 2012, 14).

Later in the same year that Gellman’s article was published, a proposal from law professor Jane Yakowitz argued instead for a broader scope of liability (Yakowitz 2011). She would not limit the application of a re-identification offence to those who explicitly undertake to subject themselves to it, but instead to any “adversary [who] discloses the identity and a piece of non-public information to one other person who is not the data producer” (Yakowitz 2011, 48). As broadened liability dramatically expands the risk of encompassing the researchers who study re-identification risk in order to strengthen, rather than compromise, the systems, Yakowitz would seek to avoid criminalizing these researchers by promoting a liability approach limiting criminal offences to wilful instances of such disclosures (Yakowitz 2011, 48–49). Appropriately carving out these exceptions has proven to be a challenging feat. Yakowitz’s approach does not seem to encompass malicious actors who disclose their re-identification method without any person’s identity itself, for example, which conceptually leads to results that are just as harmful or worse. But the dilemma is that if the offence’s scope is broadened so as to punish disclosure of re-identification methods themselves, this inescapably constitutes a direct attack on researchers.

In 2016, the third Caldicott report in the United Kingdom pushed the criminalization envelope further still (National Data Guardian for Health and Care 2016). Rather than limiting liability to intentional or even reckless acts of re-identification, in order to restore trust in anonymization, the report advocates for the adoption of “criminal penalties for deliberate *and negligent* re-identification of individuals” (National Data Guardian for Health and Care 2016, 8, emphasis added). Protection of important security research is particularly chilled when faced with broad and vague criminal prohibitions that potentially to include work to strengthen and test anonymization techniques.

Lawmakers in the United Kingdom have now taken up a similar approach. In a statement of intent regarding the legislative revisions the country will make in light of the European Union’s landmark General Data Protection Regulation, they laid out their intent to “[c]reate a new [criminal] offence of intentionally or recklessly re-identifying individuals from anonymized or pseudonymised data” whose maximum penalty will be an unlimited fine (U.K. Department for Digital, Culture, Media and Sport 2017, 10).

Law professor Jorge Contreras has proposed applying re-identification sanctions to genetic information specifically, including “civil penalties, damages, and possibly criminal prosecution,” which “would go a long way toward preventing many of the nefarious uses of genetic information that privacy advocates fear” (Contreras 2016, 46). He does not elaborate on how the preventive function would operate and on the contrary observes that a mechanism to monitor violations of the prohibition would prove elusive because the institutional entities tasked with reviewing research projects for ethics compliance “cannot be viewed as objective watchdogs of research conduct” and because the other potential monitor, namely a “governmental monitoring and enforcement function … does not exist today and is unlikely to emerge in the foreseeable future” (Contreras 2016, 49).

Other jurisdictions seeking to stem wrongful re-identification are exploring an approach that bears some similarity to that proposed by Gellman but that instead leaves criminal liability entirely behind in favour of a pure licensing arrangement between private parties.^4^ A 2014 report of France’s Senate, for example, recommends an “open data licence” that would “expressly forbid any abusive reuse that would result in removing data’s anonymity” and would include “a clause to legitimately suspend the right to reuse, and to erase or repatriate compromised data sets when a re-identification risk emerges” (Gorce and Pillet 2014, 66, author’s translation). Quebec’s data protection authority has drawn inspiration from this approach but emphasises that although licensing government data to a specific researcher offers a certain amount of protection, “the dissemination of the same data bank to the general public, in an open format, absent any limitation respecting its use obviously entails a much greater re-identification risk” and thus it suggests imposing more restrictive licenses in such cases (Commission d’accès à l’information du Québec 2016, 159, author’s translation).

Examples of recent exploration of the criminal re-identification zeitgeist are readily available in other jurisdictions, notably New Zealand (New Zealand Data Futures Forum 2014) and Canada (Office of the Privacy Commissioner of Canada 2016). The criminal re-identification offence before the Australian Parliament is addressed separately and in detail below in Part VI.

## III. Are Specific Rules Prohibiting Wrongful Re-identification Necessary?

Before assessing the implications of criminal forms of liability, it is important to address the significance of explicit rules to prevent re-identification in general.

In many respects, data protection law has remained remarkably consistent since its emergence in the early 1970s, despite ongoing revolutions in data processing and its relation to human activity that have repeatedly emerged in the interim. One of the most remarkable examples of regulatory consistency is the OECD’s publication in 2013 of a revision of its 1980 Privacy Guidelines, the latter having had a difficult-to-overstate influence on global data protection law, in which the eight data protection principles that formed the core of the initial guidelines were retained in their entirety (OECD 2013).

This is not to say that a number of significant changes have not taken place nor that a number of new data protection notions have not evolved. Breach notification rules, for example, have emerged around the world out of data subjects’ more general rights to informed consent and to information about how their data are processed. The right to erasure in article 17 of the European Union’s new General Data Protection Regulation, a less-widely accepted new data protection principle, grew out of the data subject’s right to rectification.

But explicit prohibition of re-identification is generally put forward without reference to existing data protection principles. Before adopting a new principle of its own kind, it would first be helpful to define its conceptual relationship with the existing framework.

A common—perhaps even defining—feature of data protection law, are restrictions on the collection, use, disclosure, and transfer of personal data. A general rule generally requires that such forms of processing be limited to the purposes for which the data was collected or to which the data subject has consented.

Where a deliberate attempt has been made to anonymize personal data but the attempt is legally inadequate to remove its personal character, the data remains subject to general data protection norms and malicious re-identification should thus generally constitute an improper use of personal data, as it is manifestly contrary to the purposes of the entity who attempted to anonymize it. Where an anonymization process is successful according to the legal standard, yet data is nonetheless re-identified, for example by means that could not reasonably have been foreseen, the re-identification should thus generally constitute an improper collection of what has now become personal data, as the collection was equally manifestly contrary to the purpose of anonymization.

As such, existing data protection frameworks seemingly already regulate wrongful re-identification, and new provisions may overlap with them. One potential advantage of making penalties for wrongful re-identification explicit is just that: they might serve the purpose of providing specific notice that this behaviour will not be tolerated. As an illustration, data-use agreements generally require researchers to explicitly undertake not to attempt to re-identify the data provided, and clearly an agreement prohibiting re-identification only indirectly by referring to improper “use” or “collection” of the data would be much less transparent. But it does not follow that creating a distinct prohibition on re-identification is the only, or even the best, way to notify those subject to the law that it requires that they not do so.

The potential harm in this approach, beyond adding incoherence to data protection law, is that it risks circumventing the current debate in data protection around the appropriate degree of emphasis on data use.

One recent proposal associated with Big Data Benefits argues that data protection’s traditional concern with collection, disclosure, and transfer of personal data should be dramatically reduced or abandoned altogether and that the focus should instead lie exclusively with robust regulation of improper use of personal data (Cate and Mayer-Schönberger 2013). A leading example of this approach is a 2014 White House report that argued restrictions on collection, storage, and retention are “unlikely to be scalable over time” and are damaging to Big Data (President’s Council of Advisors on Science and Technology 2014, 50). Others have argued that this shift is already a *fait accompli* in that the principle that requires that the smallest feasible amount of personal data be collected and used, “[d]ata minimization[,] is simply no longer the market norm” (Tene and Polonetsky 2013, 260).

A cousin to this approach, advocated by former Estonian President Toomas Hendrik Ilves, abandons traditional data protection altogether in favour of what he calls “data integrity.” Authorities have unrestricted access to records in the national database but with a safeguard that is built on “Blockchain-like principles” ensuring that a person’s data can be neither accessed nor modified without notifying them (Keen 2016).

Emphasis on use restrictions is resisted by privacy protection advocates. Helen Nissenbaum deems this dramatic departure from core data protection principles as “Big Data exceptionalism” (Nissenbaum 2016). The power held by data controllers over data subjects, irrespective of whether they use it, is the problem. The approach is, in fact, similar to the NSA’s redefinition of “collection” so as to exclude the control it gains of personal data until that data is looked at (Schneier 2013).

The rationale for focusing on use is also strikingly similar to that of shifting focus to improper re-identification. Despite the argument above holding that re-identification can constitute collection, outside of the idiosyncrasies of data protection law’s notion of personal data, it is essentially a use of data. Criminalization advocates argue that enacting re-identification offences would allow more widespread disclosure, collection, and transfer of ostensibly anonymized data because it would be illegal to use it in a privacy infringing way. The trend thus corresponds almost precisely with a focus on use or, if one prefers, Big Data exceptionalism.

The conscription of explicit re-identification prohibitions outside of debates around use but that nonetheless risk indirectly resolving it, should be avoided. The strongest and most apparent reason to be cautious of the prospects for protecting privacy exclusively through regulation of data uses, and a main reason that data protection has always cast its net more widely, is that the approach “is challenging to enforce because the uses of data cannot always be detected” (Naveed et al. 2015, 9), as the following two Parts explore further.

## IV. Does Criminal Prohibition Provide Greater Protection in Terms of its Scope of Application?

The discussion above in Part II of the proposals of Jane Yakowitz and of Quebec’s data protection authority raises an important factor being relied upon in support of recourse to the criminal law, namely its scope of application. For data sets that will be accessed by a limited number of researchers, contractual data-use agreement undertakings not to attempt to re-identify data may suffice, provided that effective monitoring of sanctions for breach is in place. But broad or blanket criminal liability may seem to have greater appeal to achieve deterrence where data is publicly released or made freely available for download. The same may be true where there is a risk that the data will end up in the hands of third parties to whom no data-use agreement can apply and where there may even be no way to identify a party to the agreement whose actions allowed the wrongdoer to come into possession of the data.

Another way in which the broader scope of the criminal law might promote data protection is on the question of proving harms. Plaintiff proof of tangible harm resulting from a data protection violation poses notorious difficulties in private law actions, as illustrated in the United States Supreme Court *Spokeo* case (*Spokeo, Inc. v Robins* 136 S. Ct. 1540 [2016]). The representative plaintiff in the *Spokeo* class action complained that a consumer reporting agency had built a profile on him containing inaccurate information that it then sold to a variety of users including prospective employers. The court held that irrespective of whether the company’s disclosures violated the Fair Credit Reporting Act, the complainant lacked standing to bring the claim unless he proved he had suffered a “concrete and particularized” harm as a result.

Migrating the privacy analysis to the field of criminal law eliminates the risk that privacy violators escape consequences simply because they result in harm that is difficult to legally prove, while also sidestepping the tendency of judicial decision-makers toward avoiding granting awards in damages to plaintiffs when they would amount to a windfall, with little relationship to the harm they actually suffered.

But the criminal law and its procedure may ultimately introduce more gaps in the scope of its coverage than it can close. Re-identification, for example, may be no less harmful when it is carried out by a wrongdoer who is outside the national jurisdiction where the offence is in force, yet in such cases it is even less likely to give rise to an enforceable penalty than are private law obligations.

A range of procedural and substantive safeguards also apply in the criminal context to protect the interest in liberty that further limits the scope of application. Offences based on negligence, that require no wrongful intention at all, such as that proposed in the Caldicott report (National Data Guardian for Health and Care 2016, 8), and particularly offences that do not even require voluntariness, are less likely to be ultimately enforceable, as they run contrary to longstanding criminal law protections. Similarly, each essential element of any offence will generally need to be proven beyond a reasonable doubt, rather than on a balance of probabilities, which will further reduce the situations in which a criminal sanction will ultimately be applied.

Criminal cases tend to be procedurally more complicated and thus challenging to conduct, which allow the potential to escape liability in the case of well-resourced defendants or poorly resourced prosecutors. In Europe, for example, “one needs to work with victims, data protection authorities (DPAs), police, prosecutors and criminal courts, whereas in administrative law, most roles are played by the data protection authorities (decision to investigate, decision to prosecute and decision to sanction) and the administrative courts (a posteriori controls)” (De Hert and Boulet 2016, 364).

Worse yet, the criminal law’s tendency to reject compelled self-incrimination is increasingly at odds with contemporary data protection law, which has been decisively progressing toward controller transparency in jurisdictions around the globe, notably duties to notify data protection authorities or data subjects of any data breach that they suffer. These obligations flow directly from the difficulty in even detecting improper uses of data and the fundamental importance to the integrity of any data protection regime that such undetected departures from the framework’s dictates be limited.

If data controllers are legally required to report any data protection breach, and criminal re-identification constitutes or is related to such a breach, then regimes will run afoul of the prohibition against re-identification. In some jurisdictions, the criminal sanction may then be unenforceable against the self-reporting wrongdoer.

The current trend toward heavier breach notification duties is so strong that law and policymakers even appear willing to allow them to override the possibility of imposing sanctions for the same behaviour In this vein, some have argued that criminal data protection sanctions actually strengthen breach notification rules by introducing the threat of criminal punishment only for breaches for which notification was not given (Hengesbaugh, Stoker, and Krone 2011). For reasons explored in the following part, making use of the criminal sanction in this way appears dubious, and at a minimum the implications of the privilege against self-incrimination and other scoping considerations merit careful consideration.

Yet, a final ostensible benefit of criminal sanctions over contractual arrangements between data stewards and researchers is that the criminal law is publicly visible and gives research participants knowledge of the content of their legal rights and protections and limited means to exercise them. Indeed, data subjects’ right to transparent and effective forms of administrative or judicial redress have been increasingly recognized since being affirmed in the EU Court of Justice’s *Schrems* case, which was infamous for having rescinded the determination that the U.S.–E.U. Safe Harbor framework provided adequate data protection (*Schrems v Data Protection Commissioner* (C-362/14) [2015] ECJ, ¶90). But extending rights to data subjects might be better achieved through strengthening the data-use agreements themselves, which data stewards should make transparent to data subjects, and which should ideally confer common data subject rights such rights of access, notice, redress, portability, and rectification.

## V. Does Criminal Prohibition Provide Greater Protection through More Severe Penalties?

Advocates of criminal re-identification sanctions seldom explicitly state the rationale underpinning their recourse to that particular mode of liability rather than, for example, an administrative law solution. They appear to be grounded in a mindset that envisions increasing intensity of punishment causing a corresponding increase to the prohibition’s deterrent effect and thus ensuring robust privacy protection or at least public confidence in such protection. Since the goal is maximum privacy through deterrence, their sights seem to be set on the most intense sanction that the law provides, namely criminal liability, sometimes even in its own most intense form, namely a felony or indictable offence. This corresponds with a “tough on crime” approach. Who would risk jail time just to manipulate data?

This common-sense assumption is the weakest aspect of criminal re-identification offence, as it lacks a basis in evidence, paralleling failed experiments with mandatory minimum penalties (Tonry 2009). The evidence-based cynicism of veteran experts in the field is illustrated by a leading Canadian textbook on criminal sentencing, which states in its opening sentence that “nothing we do or have done has any significant effect on the problem of crime” (Ruby, Chan, and Hassan 2012, 1), suggesting that solving such problems is never as simple as simply adopting severe penalties.

The experience of reluctant and uneven application of mandatory minimum sentences is consistent with that of criminal law offences in the field of data protection. Prosecutions are rare. The United States’ HIPAA Privacy Rule has seen fewer than two dozen criminal enforcement proceedings in its history (McGee 2015). In the European Union, “the criminal law [data protection] provisions in countries where they exist are seldom used,” perhaps because “[t]urning to criminal law implies that the data protection authorities lose control of a case” (De Hert and Boulet 2016, 365). It is neither surprising nor objectionable that they would be reluctant to turn this control over to decision-makers without data protection expertise. In countries whose data protection regimes opt for extensive criminalization, such as Belgium, in practice these proceedings bear much more resemblance to administrative law, as they privilege fines over imprisonment and data protection authorities prefer to retain control (De Hert and Boulet 2016). A rare case in Italy in which three Google executives were sentenced to six months suspended sentences for privacy violations was later overturned by the country’s Supreme Court (out-law.com 2014).

Sanctions for data misuse do, in some cases, need to be increased. For example, often the only sanction for improper re-identification of participants by researchers in existing data transfer or access contractual agreements is the possibility that the researcher will have their right to use and access the research data set revoked in the future. Although the impact this may have on their research and reputation may be more than trivial, it provides an insufficient guarantee of privacy to participants. However, to make the tremendous jump from this existing lax sanction to a felony crime would introduce a disproportionate sanction to the law that would be counterproductive to its consistent application.

In short, deterrence may be more effectively achieved by ensuring consistency, so that any violation that occurs will inescapably result in a meaningful penalty, rather than by seeking to ensure that violations have some remote chance of resulting in an especially severe penalty (Kuner 2013, 154). Criminal or regulatory norms that apply only to entities with a measure of institutional cohesion and thus accountability, and whose violations are relatively easily identifiable, such as insurers and employers who are subject the US *Genetic Information Nondiscrimination Act of 2008*, achieve much more widespread compliance than norms that apply to the population-at-large when violations cannot readily be traced to an individual or perhaps even identified, such as online sharing of copyrighted media. It is crucial not to lose sight of this potential disparity between law and practice while making policy around identifiability.

The European Court of Justice, however, appears to have done just that in its recent *Breyer* decision (*Breyer v Bundesrepublik Deutschland* (C-582/14) [2016] ECJ). The case turned on whether a set of collected dynamic IP addresses were identifiable. The Advocate General’s opinion in the case held that the literal interpretation of Recital 26 of the E.U.’s Data Protection Directive, which states that determinations of identifiability must take into account the “means likely reasonably to be used … by any other person” is too strict (¶ 64). Instead, third parties with the necessary data to re-identify the data need not be taken into account when the eventuality of it being used to that end is prohibited by law (¶ 68). The Court accepted this interpretation but found that on the facts, the existence of extraordinary legal channels prevented the data from being anonymized (¶ 68). The Court’s reasoning, however, neglects the possibility that an illegal activity might nonetheless remain widespread in practice. It is troubling for the law to deem data to be anonymized based only on a legal re-identification prohibition absent any evidence that the rule enjoys widespread adherence in practice.

Criminal re-identification penalties that target the population at large are unlikely to readily identify malfeasors, who can have relatively accessible means at their disposal to ensure their own anonymity. In advocating for such penalties, Yakowitz herself acknowledges that “detection and enforcement … would no doubt be very difficult” yet defends them on the basis that the disincentives wouldn’t necessarily “have no effect” (Yakowitz 2011, 50). Re-identification penalties that instead target a specific segment of the population, such as institutional researchers, will be generally be unable to provide greater capacity for deterrence than more targeted approaches such as undertakings in data-use agreements.

## VI. Case Study: Australia’s Proposed Re-identification Offence

Recent legislative developments in Australia may lead to a real-world test of the considerations described above.

In summer 2016, under a Creative Commons licence, the government published a research data set comprised of Medicare patient data collected between 1984 and 2014 containing a random sample of 10 per cent of patients. This data set had first undergone a “suite of confidentiality measures including encryption, perturbation and exclusion of rare events has been applied to safeguard personal health information and ensure that patients and providers cannot be re-identified” (Australian Government 2016, ¶2). The publication detailed the methodology used to encrypt patient and service provider ID numbers to allow subsequent linkage while preventing re-identification.

On 12 September, a group of academics from the University of Melbourne privately informed the government that it had been able to decrypt—in other words, to re-identify—every service provider ID in the data set. The government immediately withdrew the data set from the website where it had been posted. Although the logs indicated that it had been downloaded 1,500 times, there was no way to determine who had downloaded it, nor to whom it may have been disclosed in the interim (Middleton 2016).

On 28 September, the Attorney-General announced his intention to amend the *Privacy Act 1988* both to create a criminal offence for wrongful re-identification of such data, as well as for this amendment to apply retroactively to that same day (Brandis 2016). At least one newspaper appears to have understood the new prohibition to have already entered into force (Spooner and Towell 2016). The significance of this date is that it directly precedes the academics’ publication of their re-identification findings and general methodology (Culnane, Rubinstein, and Teague 2016a).

On 5 October, a public government database with details on 96,000 public servants was removed due to fears the “anonymized” data was re-identifiable (Towell 2016).

By 12 October, the promised amendments to the *Privacy Act 1988* had been introduced in the Senate for first reading as the *Privacy Amendment (Re-identification Offence) Bill 2016* along with an explanatory memorandum explicitly citing the U.K. Caldicott report, mentioned above in Part II of this article, as a source of inspiration. Consistent with earlier initiatives the stated objective of the bill is to take “full advantage of the opportunities that new technology creates to improve research and policy outcomes” (Parliament of the Commonwealth of Australia, Senate 2016, ¶2). Timothy Pilgrim, who is Australia’s Information Commissioner and Privacy Commissioner, publicly bolstered the bill in an article asserting that “[d]e-identification is a smart and contemporary response to the privacy challenges of data” (Pilgrim 2016, ¶7).

The bill’s four key components are, first, a new section 16CA, which lifts certain blanket exemptions from liability “due to the need for a general deterrent” that the *Privacy Act 1988* would otherwise apply to individuals and contractors. Second, section 16D criminalizes re-identification, making it an offence to intentionally re-identify data that was published by a government agency on the basis that it was de-identified for use by the general public, with a few exceptions. Third, section 16E criminalizes the intentional *disclosure* of such data even if the re-identification was itself unintentional, so long as it is known have occurred, again with a few exceptions. Finally, section 16F provides for a non-criminal penalty for failing to notify the agency of having re-identified the data, again, even if the re-identification was unintentional, so long as it was known to have occurred. Each of the criminal offences carry a penalty of imprisonment of up to two years or 120 Australian penalty units, which in this context amounts to $13,200 AUD. Section 16G appears to envision exceptions for research purposes, but these are limited to seemingly *ad hoc* exceptions approved by ministerial discretion, in consultation with the Information Commissioner.

Despite the stated intention of the bill’s drafters to avoid catching re-identification researchers within the scope of the penalty, it is unclear what is excluded. The University of Melbourne researchers warned that if the “rules had been in place in September, we might not have identified the problem in the … encryption, the data set would still be online, and the government would be unaware of its insecurity” (Culnane, Rubinstein, and Teague 2016b, ¶7). The irony of re-identification offences in general, they add, is that “[u]sually, acts that are impossible don’t need to be banned” (Culnane, Rubinstein, and Teague 2016b, ¶2).

The explanatory memorandum includes a required analysis of the bill’s compatibility with human rights, including a justification of the bill’s reverse onus on defendants requiring them to prove that one of the bill’s exceptions applies to them, which infringes on the presumption of innocence (Parliament of the Commonwealth of Australia, Senate 2016, ¶39–43) and the prohibition on retroactive criminal law (¶44–51). The memorandum failed to analyse, however, the conformity of the combination of bill’s re-identification notification duty and its criminal re-identification offence with the privilege against self-incrimination, which is incorporated in Australian law through Article 14(3)(g) of the *International Covenant on Civil and Political Rights*.

The rushed time constraints in which the legislative tests were conceived show. The explanatory memorandum needs further work, and the protections afforded to security researchers remain unclear. But these concerns are ancillary to the central question, which is whether the government objective of turning back the clock to the moment the data was thought to be robustly anonymized can be achieved or significantly furthered, through criminal re-identification offences. Can the threat of such offences really restore the privacy of those Medicare patients whose data was included in the 1,500 downloads?

For the reasons addressed earlier in this article, the proposition appears dubious. It is unclear that any occurrence of attempted or actual re-identification would be detected. Even if detected, a person sufficiently sophisticated to re-identify would quite plausibly take steps to prevent their own identification. If that wrongdoer was nonetheless identified, any of a number of procedural or substantive limits on the scope of the criminal law might frustrate a prosecution.

In sum, the notion that criminal re-identification sanctions provide legal protection that is comparable to the technical protection provided by provable anonymization is dangerously misleading.

## Conclusion

In this article, we charted developments underpinning the wave of interest in criminalizing re-identification of personal data. We raised several conceptual concerns associated with this movement, namely that it is unclear what criminalization adds to already-existing data protection tools including those described above in Part III and that it may have the perverse result of adding uncertainty and incoherence to both law and Big Data practice.

Although the strategy can initially appear to be an appealing means to dispose of a pernicious problem, by providing public perception and seeming reassurance of deterrence, it should be approached with caution. The vast majority of criminal re-identification’s proponents have addressed the topic in only a cursory manner. Even the few somewhat more sustained analyses, such as those provided by Gellman and in the Australian Bill, leave a number of the concerns and considerations raised by this article unaddressed or poorly addressed.

None of this is to say that existing approaches to data protection enforcement are adequate. To the contrary, throughout the world, enforcement likely remains the field’s least successful area (see e.g. Kuner 2013).

For the time being, as suggested by Part IV, the path to achieving consistent and reliable scope of data protection enforcement could be approached by strengthening data protection authorities’ capacity, resources, and jurisdiction to undertake enforcement action and to abandon solely complaints-based enforcement action in favour of an approach that includes proactive investigation and monitoring. In addition, enhanced monitoring mechanisms and data-subject rights in data-use agreements also merit further exploration.

Finally, as indicated in Part V, sanctions should be proportionate to deter violations and should not be practically stymied by imposing an onerous or insurmountable burden on plaintiffs to prove concrete injury. They may be community-based, where, for instance, genomic data custodians report misuse to ethics committees, the wrongdoer’s institution, or journals (Joly, Zeps, and Knoppers 2011). Regarding legal sanctions, the general approach of the new E.U. Regulation in its Article 83 provides a helpful example in this respect in prioritizing administrative fines that must be designed to be “effective, proportionate and disuasive” (¶1) and in allowing sufficient flexibility by providing for a maximum of €20 million or 4 per cent of annual revenues, whichever is greater (¶5).

Law and policy must provide appropriate redress when wrongful re-identification occurs, by deterring the behaviour and by compensating those who are harmed. But criminal sanctions appear to be a disproportionate means to this end, particularly because earnest attempts at achieving consistent enforcement through meaningful yet less drastic deterrents have generally not yet been earnestly mobilized. Finally, law and policymakers should reconsider the direction taken by the European Union’s *Breyer* decision and the proposed Australian amendments and instead treat with scepticism any approach that casts legal provisions allowing severe and blanket punishment as the functional equivalent of mathematically provable anonymization techniques, which lead data protection and Big Data down a dangerous path.

## Footnotes

1 Although the focus of this article is effectively on data protection, because the English-speaking world often uses the more vague term “privacy” even when it is essentially referring to data protection, we have referenced both concepts here. Data protection frameworks emerged from regulation of the collection, use, and disclosure of personal data that began to emerge around 1970. Privacy, on the other hand, includes a number of additional areas of the law, such as security against unreasonable search and seizure by police, the right to one’s image, duties of confidentiality, and prohibitions against eavesdropping, to name but a few.

2 This said, some scholars argue that reconciliation between data protection and Big Data benefits is possible without invoking anonymization as the bridging strategy (Laurie and Sethi 2013). For example, legal scholar Mark Taylor (University of Sheffield) has argued that confidential patient data may be used for public policy objectives if persons are provided (1) reasons to *expect* the use of data; (2) reasons to *accept* that use; and (3) that the data uses *respect* patient preferences. Taylor argues that through this “triple test” of “expect, accept, and respect,” legitimate invocations of both privacy and the public interest can serve to protect and constrain one another (Sorbie 2016).

3 And, to be precise, to others closely related to them, including their agents and employees.

4 This proposed licensing scheme is distinct from the longstanding requirement in some countries that personal data processing is made subject to an approved license for that purpose issued by data protection authorities. The proposed scheme does not involve approval from a public authority but is based instead on the idea that a private party would make data available subject to the acceptance of a license that it imposes, which would prohibit attempts at re-identification and likely provide for sanctions in the case of noncompliance with that condition.
